# Monoclonal Antibodies Application in Lateral Flow Immunochromatographic Assays for Drugs of Abuse Detection

**DOI:** 10.3390/molecules26041058

**Published:** 2021-02-18

**Authors:** Zidane Qriouet, Yahia Cherrah, Hassan Sefrioui, Zineb Qmichou

**Affiliations:** 1 Medical Biotechnology Center, Moroccan Foundation for Advanced Science, Innovation & Research (MAScIR), Rabat 10100, Morocco; qriouet.zidane@gmail.com (Z.Q.); h.sefrioui@mascir.ma (H.S.); 2 Laboratoire de Pharmacologie et Toxicologie, Faculté de Médecine et de Pharmacie, Université Mohammed V-Souissi, Rabat 10100, Morocco; cherrahy@yahoo.fr

**Keywords:** drugs of abuse, lateral flow assay (LFA), immunochromatography, monoclonal antibodies and detection

## Abstract

Lateral flow assays (lateral flow immunoassays and nucleic acid lateral flow assays) have experienced a great boom in a wide variety of early diagnostic and screening applications. As opposed to conventional examinations (High Performance Liquid Chromatography, Polymerase Chain Reaction, Gas chromatography-Mass Spectrometry, etc.), they obtain the results of a sample’s analysis within a short period. In resource-limited areas, these tests must be simple, reliable, and inexpensive. In this review, we outline the production process of antibodies against drugs of abuse (such as heroin, amphetamine, benzodiazepines, cannabis, etc.), used in lateral flow immunoassays as revelation or detection molecules, with a focus on the components, the principles, the formats, and the mechanisms of reaction of these assays. Further, we report the monoclonal antibody advantages over the polyclonal ones used against drugs of abuse. The perspective on aptamer use for lateral flow assay development was also discussed as a possible alternative to antibodies in view of improving the limit of detection, sensitivity, and specificity of lateral flow assays.

## 1. Introduction

Monitoring the use of psychotropic drugs is a public health issue and is proving to be very useful in the workplace. This is because illicit drugs can affect the cognitive and motor functions [1,2,3,4,5,6,7,8,9]. In addition, many psychoactive drugs, especially benzodiazepines (BZDs), are strongly associated with illicit drug use [10].

The concentrations of psychotropic drugs in biological matrices (saliva, blood, urine, etc.) are low, which requires very sensitive, selective, and appropriate methods for their detection and quantification [11,12,13,14,15,16,17]. The most frequently used methods of drug detection today are based on procedures for separating compounds by chromatographic standard techniques (GC, HPLC, and LC). Their quantifications are then carried out using UV, electrochemical, or fluorescence detectors. The GC-MS or, even more, LC-MS, are the most efficient methods of analysis [11,16,18,19]. However, they are expensive and require trained and qualified clinicians to conduct the analyses. Every year—in order to simplify detection methods and reduce costs—new lateral flow immunoassay (LFIA) tests appear in all fields of medicine, including cancerology, toxicology, and infectology [17,18,19,20,21,22,23,24,25,26], to be used directly in places where patients are cared for, with no specialized laboratories. The goal is to have immediate results, so that certain diagnoses can be quickly included or excluded.

The LFIA tests have several advantages over conventional diagnostic tests. They provide immediate treatments in the events of potentially life-threatening diseases, specific treatments rather than presumptive treatments, and early measures to prevent transmission of the disease (i.e., in the hospital or the community). In addition, these tests avoid unnecessary treatments and further investigation through follow-up testing [27]. Despite these advantages, each LFIA has its own characteristics, which must be known by the practitioners if they want to use it correctly.

These assays are based on the antigen (Ag)-antibody biochemical interaction and their performances depend essentially on the characteristics of the antibodies (Abs), such as affinity, specificity, production process (i.e., monoclonal Ab or polyclonal Ab), and cross-reactivity [28]. Abs are the primary reagent used for LFIA for the detection of low concentrations of analytes (the drug consumed) in the sample. Their selection (Abs) is a critical step of LFIA development. For a competitive LFIA, which is most useful in the case of small molecules, such as drugs of abuse (DOA), we used only one antibody (Ab) that was sensitive and specific to the target molecule. In the case of a sandwich LFIA, we used two Abs that could bind to the analyte with high specificity and sensitivity, but before using them, we had to test the available pairs, to determine which pair met the requirements. In general the Abs most used by the majority of the authors are monoclonal antibodies (mAbs), in which is fixed a compound (gold nanoparticles, latex microbeads, etc.) that will allow the visualization of the Ag-Abs reaction [27]. Monoclonal Abs detect only one epitope, because they are produced from a family of genetically stable cells (cloned hybridoma); consequently, they have high specificity to the Ag than the polyclonal Abs that are less specific, because they are produced by different cell clones [28].

With these considerations, this review will provide information on the production and application of mAbs directed against DOA in rapid detection test (LFIA) development. LFIA principles and designs, as well as a summary about developed LFIA to detect DOA over the past ten years, will also be discussed.

## 2. Research Methodology and Study Selection

The electronic search strategy was conducted in Google Scholar, Web of Science, PubMed, PROQUEST (Dissertations and Thesis International Database), and Scopus, in accordance with Ömer Gülpınar and Adil Güçal Güçlü guidelines published in 2013 [29]. The survey was performed using several search terms—such as LFIA test, DOA, BZDs, heroin (HRN), cocaine (COC), amphetamine (AMP), methamphetamine (MET), cannabinoids (CNB) or Δ-9 tetrahydrocannabinol (THC), psychotropic molecules, opioid or synthetic opioid fentanyl (OPI), immunochromatographic tests, mAbs production, point of care testing (POCT), aptamers, DOA detection, and DOA analysis—to select eligible studies for inclusion in the present work. A total of 139 scientific articles were used to compose the present work. The characteristics used as criteria for eligibility were:

(1) Original scientific publications from the year 2010 onwards.
(2) Studies evaluating one or more LFIA test for their ability to detect the following DOA: OPI, BZDs; HRN, THC, MET, AMP, COC, etc.

## 3. Antibodies Production Processes: Focus on mAbs against Drugs of Abuse

The Abs are glycoproteins called immunoglobulins (Ig), secreted by B-lymphocytes, components of the adaptive immune system, in response to an immunogen. There are many different isotypes or classes (IgG, IgA, IgM, IgE, and IgD), but the IgG isotype is often the major component of commercially available Abs and constitutes the most fractions of blood proteins. IgG is further divided into four subclasses (IgG1, IgG2, IgG3, and IgG4), with the numbers corresponding to the decreasing order in which they are found in the blood [30].

Abs production is simple, but there are several factors that affect the probability of an animal to produce Abs against the injected Ag (immunogen). The factors influencing immunogenicity are [31]:

- The molecular size of the injected Ags: the most active immunogens tend to have a high molecular mass (>14,000 Da). Indeed, small Ags (e.g., DOA) are known to be either non-antigenic or weakly antigenic.
- The foreignness: an antigen must be a foreign substance to the animal (not self) to elicit an immune response.
- The chemical complexity: the more complex the immunogen or substance is (chemically), the more immunogenic it will be. The DOA (BZD, heroin, amphetamine, morphine, etc.) are often of low molecular weight and, generally, for any very small Ag, the entire chemical structure is considered by the immune system as a single epitope to which an Ab binds.

Since they are unable to induce an immune response by themselves, they require a carrier molecule to act as a complete Ag. They are used as haptens or as a recognition site for the production of specific Abs by coupling them to a suitable carrier molecule (the immune response in the host animal can produce Abs against the entire immunogen and not just the drug molecule). Many proteins can be used as carriers, but the most commonly used ones are bovine serum albumin (BSA; 67 kDa) and keyhole limpet hemocyanin (KLH, 400 kDa), which are highly immunogenic because of their complexity (structure) and large sizes [32,33,34,35].

BSA is widely used as a blocking agent in development of immunoassays, such as ELISA and LFIA, because it is very accessible and available and has numerous useful groups to be linked to small molecules, including DOA, as a carrier molecule to induce the immune system. For this reason, it is recommended, for example, to use KLH as the carrier molecule (protein) to induce an immune response against the hapten and the BSA for Abs screening and purification, to assure the detection of the Ag (hapten) instead of the carrier Abs [32,33,35].

**Figure 1:**
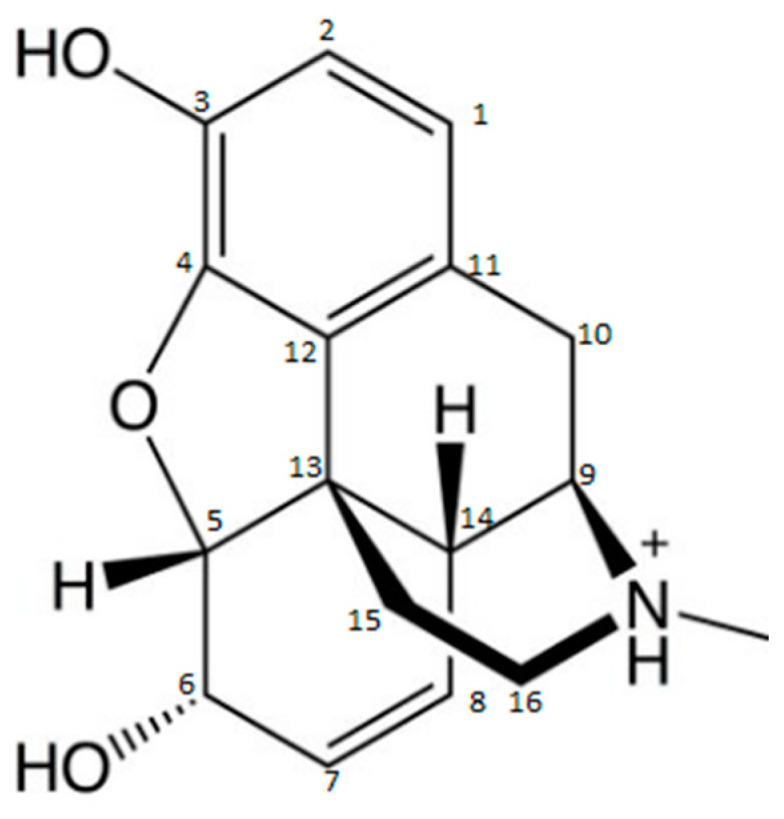
Molecular structure of morphine [36].

If morphine (MOP) is taken as a model for developing a DOA detection system based on LFIA, the carbon atoms in its positions 3, 6, 2, and group N, readily lend themselves to conjugation to the carrier protein (Figure 1).

The production of Abs directed against the MOP molecule using a carrier molecule in group N to produce a more specific assay for MOP detection is commonly used. This leaves positions 3 and 6 as antigenic determinants and, thus, allows the production of Abs more likely to be specific to MOP, without cross-reactivity to codeine (COD) or dihydrocodeine, for example. However, if the immunogen is produced via position 3, it is generally used to produce broad cross-reactive anti-opiate Abs (diacetylmorphine and MOP-3-glucuronide). Cross-reactivity to different opiates varies from one Ab to another. It is important that each Ab is fully characterized by the test developer. However, the production of MOP Abs in position 6 gives a better specificity to MOP relative to COD and MOP-3-glucuronide, and is expected to produce Abs with good cross-reactivity with 6-monoacetylmorphine and the active metabolite MOP-6-glucuronide [37].

When a mammal animal, such as a mouse, rabbit, sheep, goat, rat, or a horse (for large quantities of Ab) is immunized with an Ag immunogen, it will cause stimulation of all B-lymphocytes that produce Abs specific to that Ag. This stimulation will result in the clonal multiplication of these B-lymphocytes, which will turn into plasmacytes secreting the specific Ig. A clone produces the same Ig that has the same specificity for a given epitope. The Abs derived from a clone of plasmacytes are called monoclonal Abs (same Abs produced from a single clone).

The majority of the mAbs available in the market are IgG isotype because of their superior affinity and specificity compared to the other isotypes (IgM). However, in the natural situation, an Ag always produces a polyclonal serum [38]. Thus, the combination of Igs derived from different clones, but all recognizing different epitopes of the same Ag forms a polyclonal serum, called antiserum, specific for the given Ag.

The measure of binding strength between an Ag and an Ab is described by the affinity constant. This binding is non-covalent, reversible, and reaches equilibrium. In addition, high affinity Abs bind faster than low affinity ones and perform better in immunochemical methods [38].

In general, the commercial production of recombinant monoclonal antibodies (mAbs) follows principally similar workflow. The process begins with the generation of a mAb by immunizing an animal or by techniques using molecular biology methods involving the identification and optimization of the genetic coding sequence and the construction and identification of a stable high-producing clone.

**Scheme 1:**
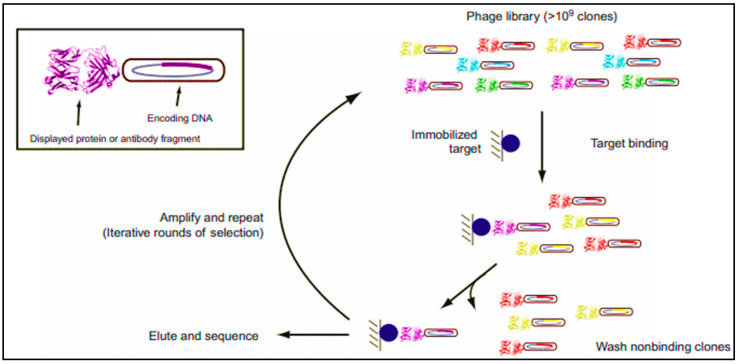
Schematic representation of phage display process. It consists of cycles, each including: incubation of the antibody (Ab) repertoire and the target, washing of nonspecific binders, elution, and amplification of specific binders for further cycle or for screening [40].

Today, in the laboratory step, several techniques are well established and commonly used to obtain mAbs, namely: Epstein-Barr virus (EBV) lymphoblastoid transformation technique, hybridoma technique, and phage display technique (Scheme 1) [39].

Phage display technique is the most commonly applied technology to produce recombinant antibodies in the laboratory settings. This helps the isolation of proteins from diverse mutagenic libraries and investigates protein-protein, protein-peptide, and protein-DNA interactions, and consists, basically, in cloning Fab coding genes into bacteriophage plasmid vectors [39]. The advantages of this methodology are multiple: one library can generate a great number of new Abs, it is an in vitro process (so animal immunizations steps are not required), and, accordingly, even toxic antigens can be tested. Moreover, the Abs molecules can be rapidly obtained [39,40,41,42]. However, for LFIA development application, mAbs produced using this technique are still not widely used, and the mostly used ones are derived from mouse hybridoma (but exhibit a downside in human therapeutics).

**Scheme 2:**
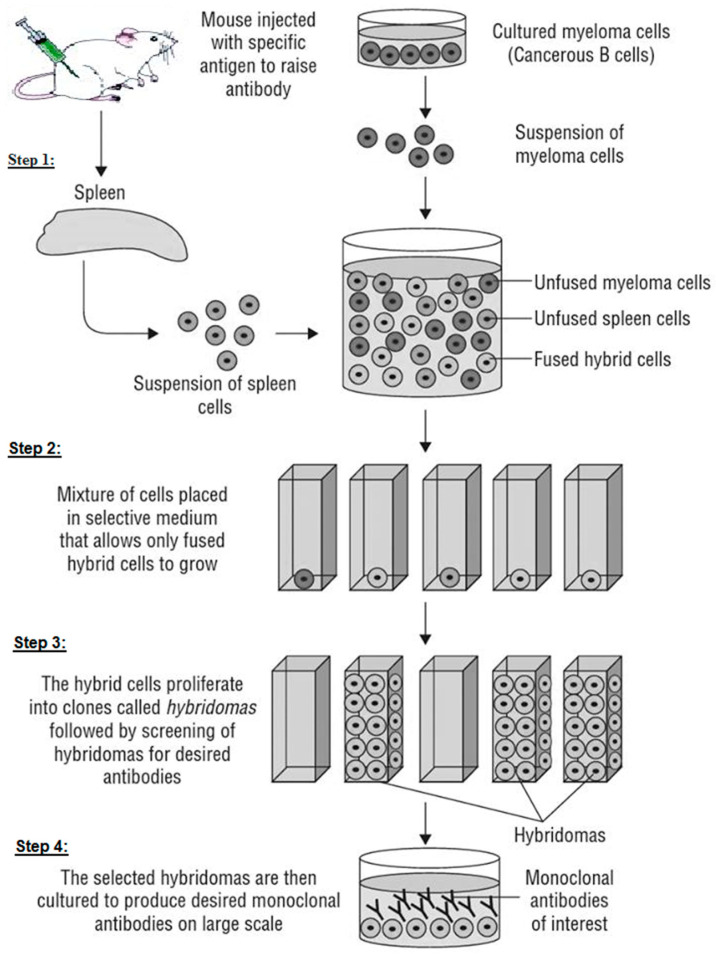
A schematic diagram showing the production of monoclonal antibodies (mAbs).

In 1975, Georges Köhler and Cesar Milstein described the first technique developed for stable monoclonal antibody production. This technique consists of creating a hybridoma, a stable hybrid cell capable of producing a single type of antibody against a specific epitope present in an antigen (Scheme 2). It is also called the technique of hybridization cell and is a method for producing large numbers of mAbs. In LFIA development application (immunoassay diagnostic or screening tests in general), it is the mostly used technique to produce mAbs in all laboratories, but has a downside in human therapeutics. The hybridoma technique is currently performed following four main steps (Scheme 2):

- Step 1: fusing the secretory lymphocyte of an Ab to the Ag used in the animal’s immunization with the myeloma using polyethylene glycol.
- Step 2: identifying the Ab secretory hybridoma.
- Step 3: isolating one cell and maintaining it in culture to obtain a single clone or family of cells, all of which are identical and secretive of the same mAb. It is limit-dilution cloning, and several successive clones are sometimes necessary to obtain a genetically stable clone.
- Step 4: growing the cloned hybridoma in a bioreactor to obtain a mAb concentrate or in a roller system to obtain the less concentrated mAb as a culture supernatant. It can be injected into the abdomen of BALB/c mice (Bagg albino, laboratory-bred strain of the house mouse) to obtain ascites-concentrated mAb.

Overall, an immunization program usually involves injecting three to six animals with the same Ag. However, if appropriate Abs are not produced after multiple immunizations, it may be necessary to repeat the program with different animals and possibly a different immunogen [37].

This method was used by several authors for the development of mAbs against DOA. Indeed, Dehghannezhad et al. in 2012 [43] used it to produce a mAb and conjugated it to gold nanoparticles (GNPs) to develop a rapid competitive immunochromatographic strip test to detect MOP in urine samples. It was also used to develop Abs for the diagnosis and screening of different diseases and clinical cases, including arthritis, breast cancer, psoriasis, leukemia, transplant rejection, asthma, and toxicity [44,45,46,47,48,49,50,51,52].

## 4. Performances of an Antibody

After the Ab is produced, as described before, surface plasmon resonance (SPR), equilibrium dialysis, ELISA, or many other methods are widely used to indicate its affinity (termed as binding strength or binding constant) to the Ag, and demonstrate its characteristics and binding to the target drug in real-time, and in a label-free manner, using a refractive index change at a metal surface [53,54]. There is also the possibility of using ELISA to verify that the Ab meets the need with the target drug (i.e., sensitivity, specificity). In this way, the binding and displacement can be observed with each Ab. Careful titration of the Abs and labeled drug derivative may improve the assay characteristics, and then the assay may be further optimized by the addition of other proteins, surfactants, and stabilizers to the assay buffer.

### Applications of mAbs and Their Comparison with Polycolonal Antibodies in the Development of LFIA

Specific mAbs provide accurate testing. They are used for the determination of ABO and rhesus blood groups, for HLA tissue grouping, for the immunolabeling of acute leukemia and for the development of immunological tests (enzyme-linked immunosorbent assay (ELISA), lateral flow immunoassay (LFIA), radioimmunoassay (RIA), etc.) [18,19,20,21,27,31,55,56,57,58,59,60]. Other Abs issued from animals conjugated to markers or enzymes are used for diagnostic kits manufacturing, immunocytochemical analysis, and research [18,19,20,21,27,31,55,56,57,58,60,61,62].

Their frequent usage in basic research has led to the study and the understanding of many biological processes. Moreover, a panel of mAbs is usually used to map and study the role of epitopes in certain cellular functions and mechanisms. They have also an important role in proteomics and mass biological screening tests [18,19,20,21,31,43,55,56,58,59]. In general, mAbs are used in diagnostic, agri-food, veterinary, microbiological, and toxicological tests [20,21,31,56,58,63]. However, mAbs generally have less affinity than pAbs, which may lead to less sensitive assays. It should be noted that in drug detection tests, an Ab may be too specific as it may be desirable to have broad cross-reactivity with a family of drugs (such as BZDs) or with a single drug and its metabolites (such as buprenorphine).

The mAbs offer the advantages of purity and homogeneity, which is useful in the circumstances where the Ab is labeled or conjugated within the framework of the LFIA’s development [61,64,65]. They all recognize specifically a single epitope and are homogeneous compared to pAbs, which allows the testing to be standardized. The monospecificity provided by mAbs, makes it possible to understand and evaluating changes in molecular conformation and structure, phosphorylation states, protein-protein interactions, and in identifying single members of protein families. However, the monospecificity of mAbs may also limit their advantages, because they should be generated to the Ag epitope to which it will bind (small change in the structure of an epitope can affect the function of a mAb) [38,66]. They can also identify an antigenic determinant in complex mixtures, such as biological fluids (blood, urine, milk, saliva, etc.).

Both polyclonal and monoclonal antibodies have their own advantages and disadvantages, which make them useful for different applications. The debate regarding whether mAbs are better than pAbs has been raging for years. Some researchers praise the batch-to-batch consistency and single-isotype nature of monoclonals, others swear by the ability of polyclonals to work in a wider range of applications, often enabling detection of the target antigen in both its native and denatured states.

The pAbs are heterogeneous and have a wide specificity than mAbs, because they are produced by a large number of B cell clones, each generating pAbs to a specific epitope. The pAbs’ production techniques are easy, fast, and low-cost compared to mAbs’ production techniques. However, their production costs depend on the quantities required and their use.

The best use of pAbs is to detect unknown antigens. pAbs are used as a secondary antibody (detectors) in immunoassays (e.g., ELISA, western blotting, microarray assays, immunohistochemistry, flow cytometry). Their role is to bind to different epitopes and amplify the signal, leading to better detection. When pAbs are used as detectors, more steps are needed, such as labeling and affinity purification, which may increases the costs. Moreover, a production of large quantities of PAbs requires a large number of animals, restrictive farming conditions, and expensive infrastructure.

In contrast, mAbs are often used as primary antibodies in immunoassays because of their ability to bind specifically to a single epitope of an Ag. They are easy to label and provide an unlimited source of antibody that is homogeneous and, once characterized, predictable in its behavior. Nowadays, mAbs specificity can be expanded by combining multiples mAbs that lead to the capture of multiple epitopes of an Ag.

Another advantage of mAbs is that, once their line is established, their supply is infinite, and the risk of isolating the desired cell line never has to occur again. In the opposite way, pAbs are prone to batch-to-batch variability and there is no guarantee that immunizing other animals will yield to a useable Ab.

## 5. Lateral Flow Immunoassays (LFIA)

### 5.1. Basic Components of a LFIA

The LFIA is a simple to use device used to confirm the presence or absence of a target analyte; also known as, quick test, lateral flow device, immunochromatographic assay, or as lateral flow assay (LFA) that is applicable to point-of-care testing (POCT). The principle of an LFA is based on the movement of a liquid sample though a strip with attached molecules that interact with the analyte, providing a signal that can be visually detected. Although the principle behind the LFA is simple, the device has a complex architecture, and many critical elements need to be considered during instrumental design.

**Figure 2:**
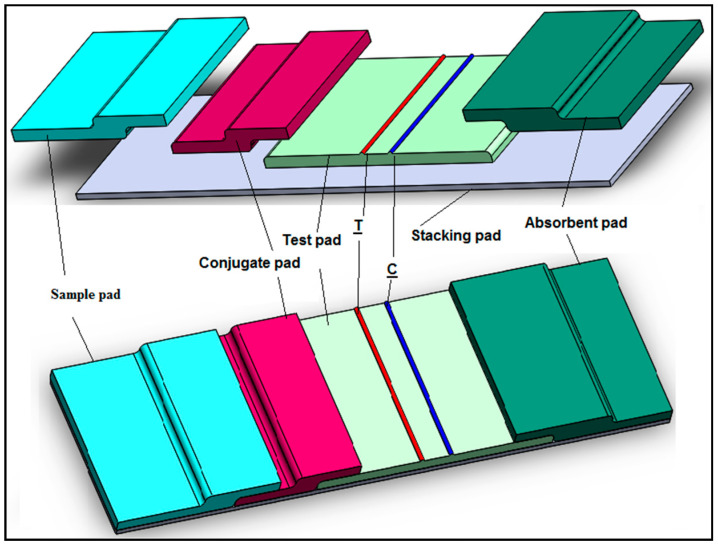
Schematic representation of a typical lateral flow assay (LFA) that is composed of four types of membrane.

Most of these listed tests are presented in the form of a plastic cassette or a card that contains nitrocellulose strips visible through the different windows or cells of the cassette [20,27,67,68,69,70,71]. In general, membrane-based tests consist of overlapping membranes or pads made of different materials that are mounted on a backing card, as described below (Figure 2).

#### 5.1.1. Sample Pad

The sample pad is a membrane in which the sample is deposited, distributed, then directed to the conjugate pad (Figure 2). It is generally made of cellulose fiber with a good bed volume and low-analytes binding, to avoid their loss and ensure a good distribution of them. According to the sample biological matrices (saliva, water, blood, urine, serum, feces, milk, etc.), a sample pad pretreatment may be necessary before dispensing the sample. This pretreatment using some proteins, such as bovine serum albumin (BSA), buffer salts, or other liquids, is necessary to optimize the pH and the sample viscosity, to control the flow rate and also to avoid nonspecific binding [20,27,67,70,71,72]. Moreover, the sample pad is used as filter in order to remove redundant materials (e.g., red blood cells).

#### 5.1.2. Conjugate Pad

This membrane is composed of cellulose filters or glass fibers. The main role of the conjugate pad is to hold the dried biorecognition elements (Abs, targets molecules or Ags) coupled to a particulate label (gold nanoparticles, colored latex, and carbon) and keep them functional and stable during the performance of the test (Figure 2). This is ensured by the conjugate buffer that contains carbohydrates (such as sucrose and borate), which serves also as a preservative and a re-solubilization agent. To select a conjugate pad, we should take into consideration the following points:

- Low non-specific reaction of the sample or the antibody coupled to a nanoparticle (label) (Ab-NP).
- Release of the Ab-NP or the sample should be quick and consistent between individual test strips.
- Ab-NP must remain functional when dried on it.

After the selection of the appropriate conjugate pad, to achieve a high sensitivity of the LFIA we should pretreat it to minimize non-specific binding by blocking protein binding sites (using BSA solutions) and control the pH [20,27,67,70,71,72].

#### 5.1.3. Test Pad, Reaction Membrane, or Nitrocellulose Membrane

The test membrane (Figure 2) is made of commonly materials such as nitrocellulose, polyvinylidene fluoride, cellulose acetate, or polyether sulfone. This membrane is considered as the most critical element in LFA strips that allows to read and to interpret the results. In order to select the appropriate test pad, it is important to consider the capillary flow time (FT) defined as the time a liquid (sample) needs to migrate along a membrane expressed in seconds/centimeters. This is the more accurate parameter, and it should be used when selecting the most effective strip material. Moreover, membrane pores size as well as the binding efficiency are a crucial criterion for test pad selection.

In general, the test membrane contains two lines named T for test and C for control (Figure 2). During the test performance, the liquid sample migrates and interacts with the analytes that are bound in the T and the C lines. The C line consists of testing the performance and control the reaction of the LFIA. The longer the membrane is, the more it allows a longer interaction between the Ab-NP and the analytes to be detected at the test line (T) [20,27,67,70,71,72].

#### 5.1.4. Absorbent Pad or Wick Pad

The absorbent pad (AP) (Figure 2) is generally composed of a cellulose fiber that can decrease the FT by hindering the backflow [73,74,75], absorbing and increasing the volume of the sample through the entire assay (LFIA) and reserves waste. In addition, the right AP selection can increase the performances of the test and the criteria for selection are the same as that of the sample pad (material, thickness, etc.) [20,27,67,70].

There are many membranes available from multiple commercial sources, the selection of the appropriate one, as well as developing the right treatment plan, is essential for any LFIA.

Generally, the criteria for selecting a membrane for a LFIA to check are:

- The analytes (drugs or proteins, etc.) size and the sample liquid viscosity.
- The porosity and the pore size.
- The thickness (µm) of each membrane.
- The potential coating or treatment that the membrane surface needs.

### 5.2. Labels

Several molecules can be used for the revelation step namely: colloidal gold (GNPs); that is a colloidal suspension of nanoparticles of gold in water, liposomes, magnetic bead, silver nanoparticles, colored latex beads, quantum dots, organic fluorophores, magnetic nanoparticles (MNP), textile dyes, carbon nanoparticles, selenium nanoparticles, up converting phosphors, enzymes, and others [20,21,27,61,64,65,72,76,77]. These marked molecules must be able to retain their physicochemical properties after coupling to any Ab or nucleic acids and must also be detected in small quantities (ng) (concentrations in biological matrices) [57,61,78,79]. A reaction must also be revealed in the control window (C) (sandwich format) (Figure 2), regardless of the test window (T) result, to ensure that the procedure goes smoothly, and the test runs correctly.

### 5.3. Formats of LFIA

**Scheme 3:**
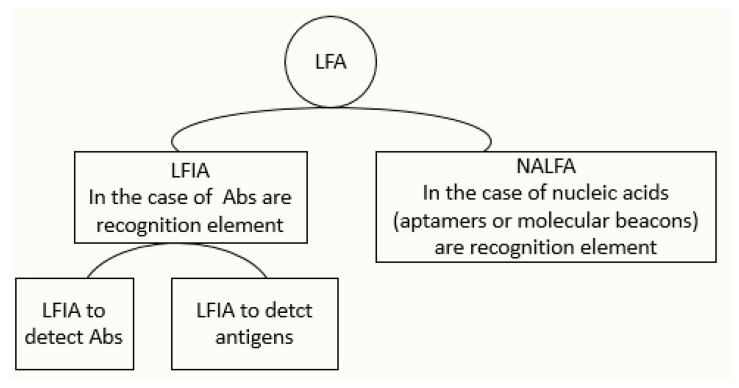
Classification of LFA according to the element of revelation.

According to the element of revelation or detection, the LFAs are classified according to the following Scheme 3 [20,21]:

Nowadays, in the DOA field, there are a few commercialized kits based on the nucleic acid lateral flow assays (NALFA) or nucleic acid chromatographic tests. The most well-known and commercialized ones are the LFIA or immunochromatographic tests, where the revelation is often done by an Ab [20], based on Ab-Ag reaction [20,21,61,76,80].

**Figure 3:**
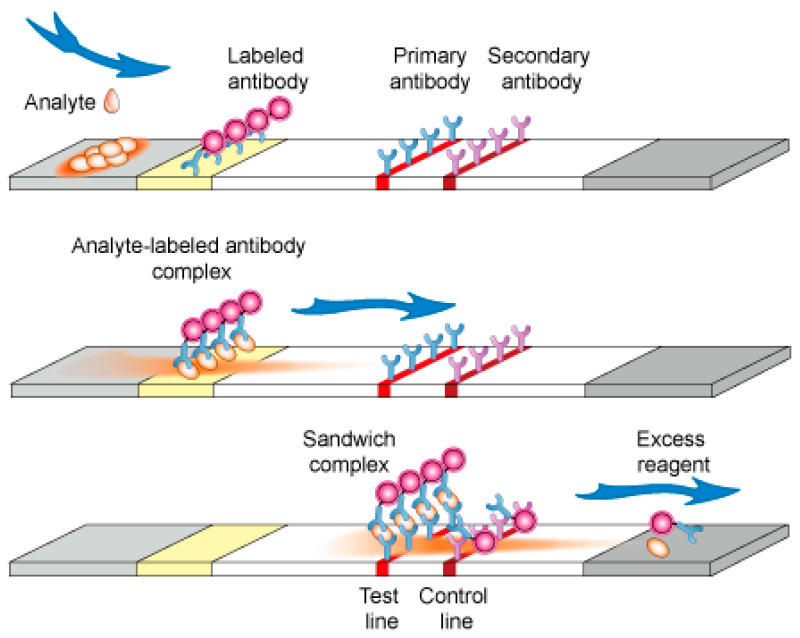
Sandwich immunochromatographic test [84].

**Figure 4:**
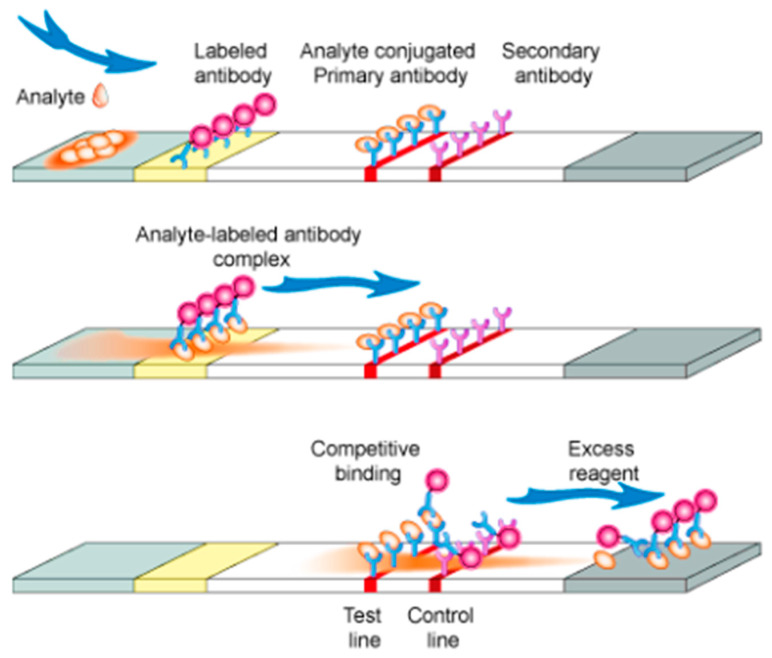
Competitive immunochromatographic test [84].

Although, depending on the nature of the molecule sought, there are essentially two types of LFIA (Scheme 1): those based on Ag detection (e.g., BZDs, AMP, etc.) [57,61,80] and those based on Ab detection (e.g., HIV infection, Chagas disease or polyarthritis) [80,81,82]. Likewise, according to the size of the element detected (Ag or Ab), and the number of their detectors, there are tree common formats of the LFIA described below (Figure 3 and Figure 4).

#### 5.3.1. Sandwich Format

The sandwich format is developed for the detection of large molecules, such as human serum albumin or Abs that have more than two epitopes or binding sites (HIV, arthritis, etc.) [80,81,83] (Figure 3).

In a sandwich assay, the T and C lines contain both the target molecule Abs. Furthermore, another Ab against the same target molecule is conjugated to the nanoparticles (Ab-NP) to reveal the presence of the analyte. The results interpretation is simple. The signal is proportional to the concentration of analytes in the sample. If the target molecule is present in the sample, the Ab-NP will bind to it at the test line (T) and give a high signal intensity. In the opposite case, the Ab-NP will not bind to the Abs in the T line and no signal will be revealed (Figure 3).

#### 5.3.2. Competitive (or Inhibition) Format

The competitive format is usually very useful for small molecules, such as psychotropic drugs (e.g., BZDs) [20,43,80] (Figure 4).

The signal intensity is inversely proportional to the concentration of the target molecule present in the sample (Figure 4); it operates on the same principles as the competitive enzyme-linked immunosorbent assays (cELISA). The T line contains the target molecules (e.g., DOA) fixed. When the target molecule is absent in the sample, unbound Abs coupled to NP (Abs-NP) will bind to these target molecules (fixed on T line), which show a visual coloration. Conversely, when the target molecule is present in the sample, it binds to the Abs-NP and stops their binding to the target molecule (fixed) in the T line, so no visual coloration will appear in the T line, or it will show some colors, but the signal is lower if an analyte is present.

#### 5.3.3. Complex Format or Multiplex Format

This test format can detect simultaneously more than one type of molecules [60,85,86]. The test contains a number of T lines equal to the number of target analytes to be detected. Recent works showed that, in many LFIA, combining several analytes into panels is critical for enhancing the diagnostic precision for a specific addiction or a disease, improving diagnostic efficiency and reducing cost [60]. Guteneva NV and al [61] developed a lateral-flow assay based on the quantification of MNP labels for multiplexed immunodetection of MOP, fentanyl, and MET in urine samples. The MNP used for LFIA in the literature vary in size (10–400 nm), they could be used for qualitative or quantitative measurements if they are coupled with an external reader, as well as enable immunomagnetic separation, which improves selectivity and sensitivity [87].

#### 5.3.4. The Performance of a LFIA (Validity of the LFIA)

In general, a screening test is used to screen within a healthy target population those likely to have a disease from those healthy. This ability depends both on the test’s own performance and on the characteristics of the tested population. In general, a screening test should be inexpensive, simple to perform, valid, reliable, reproducible, and acceptable [17].

The specific performance of a screening test is its sensitivity and specificity [17,88]; they define the intrinsic validity of the test. They are defined and calculated under experimental conditions and are, therefore, independent of the type of person tested and the population studied.

In the DOA field, the characteristics of the population tested, in particular the prevalence of consumption of the target molecule (DOA), influence the extrinsic performance of the test. These extrinsic performances are the positive (PPV) and negative predictive values (NPV). They are relatives to the use of the test for a given population and differ according to the characteristics of the tested population. They are defined and calculated in the context of the screening situation and make it possible to assess the relevance of the use of the test in this specific population.

#### 5.3.5. Intrinsic Performances: Sensitivity and Specificity

The sensitivity of a given test is the probability that this test will be positive if the person is consuming a DOA.

The specificity of a given test is the probability that the test will be negative if the tested person is not consuming a DOA.

The sensitivity of the test can therefore be calculated according to the following formula [88]:

Sensitivity = True positives (TP)/(TP + False negatives (FN)) × 100,(1)

The specificity of the test can be calculated according to the following formula:

Specificity = True negatives (TN)/(TN + False positives (FP)) × 100,(2)

The sensitivity and specificity of a test are interrelated. The increase in the sensitivity of a test is always at the expense of its specificity and vice versa.

The following Table 1 shows the likely results of LFIAs. The current conditions of the subjects (consumer or non-consumer) are presented in columns in terms of the test results in rows (positive or negative).

However, a test is accurate if the number of false positives (FP) and false negatives (FN) is the lowest possible.

#### 5.3.6. Extrinsic Performance: Positive (PPV) and Negative Predictive Values (NPV)

The PPV and NPV are influenced by the prevalence of the consumption of a target molecule in the tested population. If the test is done in a population with a high prevalence, it is more likely that people whose test result is positive have the disease than if the test is done in a population with a low prevalence of the disease (which is usually the case in screening tests). These two values can be calculated according to the following formulas:

PPV = TP/(TP + FP) × 100,(3)

NPV = TN/(TN + FN) × 100,(4)

However, a screening test must offer a good compromise between high sensitivity (to screen the largest number of suspicious people) and high specificity (to avoid using the confirmatory test in healthy people).

### 5.4. Limitations and Opportunities in LFIA

The LFIA are easy to use, provide rapid and low-cost tests, samples do not need pretreatments before the analysis, and they have a long shelf life.

However, LFIA are considered as screening tests and they have some limitations: they cannot be used for quantification and, sometimes, their results need to be confirmed (especially when using samples with a high level of molecules that present some structural similarities to target analytes), further using standard and independent techniques, such as LC-MS-MS, GC-MS, HPLC, ELISA, RIA, PCR, etc. [11,17,89,90,91,92,93,94,95]. In routine, different compounds may present some structural similarities, which can generate false positive results due to Abs cross-reactions. For example, the same result is obtained when analyzing cannabis in a sample from a person taking drugs containing niflumic acid. All of the commercial tests available, whether salivary or urinary, present the same risk of errors. However, some are much more reliable than others, and that depends, as said above, on the characteristics of the Abs used as the detection molecule.

Various LFIA tests (competitive ones) have already been described for the specific or simultaneous detection of psychotropic molecules. In general, the tests with a specificity and sensitivity close to 100% are considered good tests. Their use is easy to implement in any analytical toxicology laboratory or in the workplace.

The results of our study selection research from 2010 to 2020 were reported in Table 2. In these reports, the test used is a competitive LFIA. The confirmatory methods, the used sample, the analytes, the labels and conjugates, and the limits of detection of the tests (LODs) are reported below (Table 2). In general, the analysis time is from 5 to 15 min.

## 6. Perspectives

In recent years, the concept of aptamers, artificial Abs, or “intelligent ligands” has been introduced. Aptamers are synthetic oligonucleotides (DNA or RNA), selected and characterized by systematic evolution of ligands by exponential enrichment (SELEX) [104,105,106,107], based on their high affinity and specificity to target Ags, such as peptides, drugs, proteins, small molecules cells, and tissues.

These molecules can be used as a possible alternative to mAbs in biomedical research, since they have affinities and specificities comparable to those of Abs for various molecules [105,108,109,110,111,112,113,114,115,116,117,118,119,120,121,122,123,124].

Aptamers can also be used for targeted therapeutics or drug discovery [125,126,127,128,129,130,131,132,133,134,135,136], because they are non-immunogenic and can be easily stabilized by chemical modifications. As far as chemical and biological properties are concerned, their use against mAbs has several advantages. They are able to regenerate and to mark, stable to ambient temperature with a high reproducibility, and their structure can be modified. Moreover, their production costs can be reduced since they can be obtained by chemical synthesis, avoiding the use of animals or cells.

Nowadays, the aptamers are not widely used in LFAs. However, for the detection and recognition of psychotropic molecules, few aptamers were identified, more being devoted to COC, or its metabolite benzoylecgonine (BE), AMP, or MET (Table 3). Moreover, there are some aptamers for toxins detection, such as ochratoxin A (OTA) (Table 3). Since then, refined sequences have been identified and used to detect DOA (Table 3). These refined sequences are promising and require more studies to validate the use of aptamers for the detection of psychotropic drugs.

## 7. Conclusions

Monoclonal Abs have allowed the development of highly sophisticated assay methods that detect and quantify numerous markers and molecules in biological matrices, with a significantly improved specificity compared to polyclonal Abs. They are still widely used in some immunoassay tests, in particular because of their high sensitivity and specificity.

The contribution of Abs to human development holds great promise for the future. Their usefulness is already proven in the new technologies in genomics, proteomics, imaging, and nanobiotechnology. All LFIA tests require the use of Abs; it is this element of the system—and how it is produced—that is the key to the test’s performance (sensitivity and specificity).

The discovery of the aptamers opens up prospects with great socioeconomic impacts. It is clear that the acquisition of these technologies by laboratories is of particular importance for the future of the pharmaceutical industry. Indeed, investments must be made in this area.

## Figures and Tables

**Table 1:** Probable results for each test (lateral flow immunoassay, LFIA).

Test Results	DOA Consumption		Total (n)
	Consumer	Non-Consumer	
Positive (P)	TP	FP	Total P tests
Negative (N)	FN	TN	Total N tests
	Total of consumers	Total of non-consumers	total numbers

True positives (TP) are the set of consumer subjects with a positive test result. True negatives (TN) are the set of non-consumer subjects with negative test result. False positives (FP) are those subjects who are not consumers, but their test result is positive. False negatives (FN) are subjects who are consumers, but their test result is negative.

**Table 2:** LFIA methods for the determination of drugs of abuse (DOA) in biological samples that have been reported in the literature (from 2010 to 2020).

Reference	Molecules Detected (Analytes)	LOD (ng/mL)	Confirmatory Methods	Used Sample	Molecules of Revelation (Labels)
[43]	MOP	2000	TLC *	Urine	GNPs
[96]	COC	5	ELISA **		MB
[61]	MOP	0.60	Not given		MNP
	MET	3			
[76]	MOP	20	LC-MS ***	Saliva	Up-converting phosphor technology
	MET	25			
[97]	MOP	1	Not given		GNPs
[79]	THC	0.01			Fluorescent and Gold Particle
[57]	THC	1–10	UPLC-MS/MS ****		Not given
		1–5		Serum	
[98]	OPI	100	Not given	Saliva	
		8		Urine	
[20]	BZDs (Diazepam)	Not given		PBS	GNPs
[96]	COC	5	ELISA	Urine	Magnetic bead
[99]	THC	0.19	LC–MS-MS	Sweat	Not given
	BZD	0.09			
	MOP	0.068			
	AMP	0.08			
[100]	Fentanyl (FTY)	1	LC-MS/MS	Urine	GNPs
[101]	6-monoacetylmorphine	4	UPLC	Saliva	GNPs
[102]	MET	125	LC-MS	Blood	GNPs
	AMP	125			
	MOP	50			
	COD	50			
	Dihydrocodeine	50			
	Diazepam	25			
	Alprazolam	60			
	Estazolam	15			
	Prazepam	75			
[103]	MOP	0.1	Not given	Chafing dish condiments	GNPs

LOD: Limit of detection. * TLC: Thin-layer chromatography. ** ELISA: enzyme-linked immunosorbent assay. *** LC-MS: Liquid chromatography tandem mass spectrometry. **** UPLC-MS/MS: Ultra performance liquid chromatography-tandem mass spectrometer.

**Table 3:** Some nucleic acids LFA methods for the determination of DOA in biological samples that have been reported in the literature.

References	Molecules Detected	LOD	Used Sample	Confirmatory Methods	Aptamer Used
[113]	AMP or MET	0.82 μM	Urine	Not given	5′-ACG GTT GCA AGT GGG ACT CTG GTA GGC TGG GTT AAT TTG G-3′
[112]	COC	0.138 nM	Artificial urine	HPLC	5′-C6-NH2-AGACAAGGAAAATCCTTCAATGAAGTGGGTCGSH2-C3-3′
	BE	1.66 μM			
[55]		725.27 ± 3.17 ng/mL			5′-C6-NH2-AGACAAGGAAAATCCTTCAATGAAGTGGGTCG-SH2-3′
		741 ± 1.28 ng/mL	Synthetic Saliva		
[111]	COC	5 ± 8.9 μM (1.5 ± 2.7 μg/mL)	Acetonitrile	Not given	5′-GGGA GAC AAG GAA AAT CCT TCA ATG AAG TGG GTC GACA-3′ 5′-GAC AAG GAA AAT CCT TCA ATG AAG TGG GTC-3′
[137]		293 pM	Rat serum	Not given	5′-CCATAGGGAGACAAGGATAAATCCTTCAATGAAGTGGGTCTCCC-Thiol-3′ 5′-FAM ATTGAAGGATTTATCCTT GTCTCCCTATGCTTCAAT-Biotin-3′
[138]		5.0 ± 0.1 pmol/L	Human blood serum	Not given	5′-C6-NH2-AGACAAGG AAAATCCTTCAATGAAGTGGGTCG-SH2-3′
[139]	OTA	1 ng/mL	Red wines	ELISA	5′-GAT CGG GTG TGG GTG GCG TAA AGG GAG CAT CGG ACA AAA AAA AAA AAA AAA AAA-SH-3′
